# Antifibrotic Effects of Amyloid-Beta and Its Loss in Cirrhotic Liver

**DOI:** 10.3390/cells9020452

**Published:** 2020-02-17

**Authors:** Gayane Hrachia Buniatian, Ralf Weiskirchen, Thomas S. Weiss, Ute Schwinghammer, Martin Fritz, Torgom Seferyan, Barbara Proksch, Michael Glaser, Ali Lourhmati, Marine Buadze, Erawan Borkham-Kamphorst, Frank Gaunitz, Christoph H. Gleiter, Thomas Lang, Elke Schaeffeler, Roman Tremmel, Holger Cynis, William H. Frey, Rolf Gebhardt, Scott L. Friedman, Wolfgang Mikulits, Matthias Schwab, Lusine Danielyan

**Affiliations:** 1 Department of Clinical Pharmacology, University Hospital of Tübingen, 72076 Tübingen, Germany; schwinghammer.ute@gmail.com (U.S.); email.martinfritz@googlemail.com (M.F.); barbara.proksch@uni-tuebingen.de (B.P.); michael.glaser@uni-tuebingen.de (M.G.); alilourhmati@yahoo.de (A.L.); buadze@hotmail.com (M.B.); christoph.gleiter@med.uni-tuebingen.de (C.H.G.); Matthias.Schwab@ikp-stuttgart.de (M.S.); 2 H. Buniatian Institute of Biochemistry, National Academy of Sciences of the Republic of Armenia (NAS RA), Yerevan 0014, Armenia; seferyant@yahoo.com; 3 Institute of Molecular Pathobiochemistry, Experimental Gene Therapy and Clinical Chemistry, RWTH University Hospital Aachen, 52074 Aachen, Germany; rweiskirchen@ukaachen.de (R.W.); eborkham@ukaachen.de (E.B.-K.); 4 Children’s University Hospital (KUNO), University of Regensburg, 93053 Regensburg, Germany; Thomas.Weiss@klinik.uni-regensburg.de; 5 Department of Neurosurgery, University Hospital of Leipzig, 04103 Leipzig, Germany; Frank.Gaunitz@medizin.uni-leipzig.de; 6 Dr. Margarete Fischer-Bosch Institute of Clinical Pharmacology, 70376 Stuttgart, Germany, and University of Tuebingen, 72076 Tuebingen, Germany; Dr.Thomas.Lang@gmx.de (T.L.); Elke.Schaeffeler@ikp-stuttgart.de (E.S.); Roman.Tremmel@ikp-stuttgart.de (R.T.); 7 Department of Drug Design and Target Validation, Fraunhofer Institute for Cell Therapy and Immunology, 06120 Halle, Germany; holger.cynis@izi.fraunhofer.de; 8 Center for Memory & Aging, HealthPartners Neuroscience Center, St. Paul, MN 55130, USA; alzheimr@umn.edu; 9 Rudolf-Schönheimer Institute of Biochemistry, Faculty of Medicine, University of Leipzig, 04103 Leipzig, Germany; ivs.gebh@t-online.de; 10 Division of Liver Diseases, Icahn School of Medicine at Mount Sinai, New York, NY 10029-6574, USA; Scott.Friedman@mssm.edu; 11 Department of Medicine I, Institute of Cancer Research, Comprehensive Cancer Center, Medical University of Vienna, Vienna 1090, Austria; wolfgang.mikulits@meduniwien.ac.at; 12 Department of Pharmacy and Biochemistry, University of Tuebingen, 72076 Tuebingen, Germany; 13 Departments of Biochemistry and Clinical Pharmacology, and Neuroscience Laboratory, Yerevan State Medical University, Yerevan 0025, Armenia

**Keywords:** liver cirrhosis, liver sinusoidal permeability, liver sinusoidal endothelial cells, hepatic stellate cells, beta secretase, presenilin, endothelial nitric oxide synthase, myelin basic protein, Alzheimer’s disease, neprilysin, astrocytes

## Abstract

The function and regulation of amyloid-beta (Aβ) in healthy and diseased liver remains unexplored. Because Aβ reduces the integrity of the blood-brain barrier we have examined its potential role in regulating the sinusoidal permeability of normal and cirrhotic liver. Aβ and key proteins that generate (beta-secretase 1 and presenilin-1) and degrade it (neprilysin and myelin basic protein) were decreased in human cirrhotic liver. In culture, activated hepatic stellate cells (HSC) internalized Aβ more efficiently than astrocytes and HSC degraded Aβ leading to suppressed expression of α-smooth muscle actin (α-SMA), collagen 1 and transforming growth factor β (TGFβ). Aβ also upregulated sinusoidal permeability marker endothelial NO synthase (eNOS) and decreased TGFβ in cultured human liver sinusoidal endothelial cells (hLSEC). Liver Aβ levels also correlate with the expression of eNOS in transgenic Alzheimer’s disease mice and in human and rodent cirrhosis/fibrosis. These findings suggest a previously unexplored role of Aβ in the maintenance of liver sinusoidal permeability and in protection against cirrhosis/fibrosis via attenuation of HSC activation.

## 1. Introduction

The generation and degradation of amyloid-beta (Aβ) in the central nervous system (CNS) are well characterized, but these pathways are poorly understood in the liver. Although the liver generates, takes up, and degrades Aβ peptides [1], little is known about its role in healthy and cirrhotic liver. In progressive fibrosis leading to cirrhosis, activated hepatic stellate cells (A-HSC) are the primary fibrogenic cell type following their transdifferentiation from a quiescent, vitamin A-rich cell type to myofibroblasts (reviewed in [2]). Interestingly, A-HSC acquire many features resembling healthy brain astrocytes [3,4], which protect against Aβ-induced toxicity in the CNS [5]. Therefore, we examined whether HSCs, like astrocytes, contribute to the homeostasis of Aβ.

Aβ is generated from its parent molecule, the amyloid precursor protein (APP), through sequential cleavage by three specialized proteases: α-, β- (BACE1), and γ-secretase complex containing presenilin (PS) 1/2 [6]. Cleavage of APP through α-secretase is the major physiological nonamyloidogenic route of APP maturation, resulting in the release of a soluble 100–120-kDa *N*-terminal fragment (βAPPsα) and a small *C*-terminal membrane-bound segment (C83). Cleavage of APP by β-secretase (BACE), a membrane bound aspartic protease initiating the first step of amyloidogenic APP degradation, produces large soluble APP-beta fragment (sAPPβ) and 99-amino-acid beta-carboxyterminal APP fragment (C99, β-CTF). γ-secretase cleaves C99 mainly after residue 40 and partly after residue 42, thus generating the peptides Aβ40 and, to a lesser extent, Aβ42, as well as other Aβ fragments that vary in length and hydrophobicity [6,7]. High heritability of mouse presenilin 2 has been demonstrated in the liver, but not the brain [8], indicating that liver is a powerful source of Aβ peptides. Interestingly, in the liver of Alzheimer’s disease (AD) patients, the total Aβ level is 1/8th that found in the liver of a nonaffected control group [9], suggesting that there is either reduced production or accelerated mechanisms of hepatic Aβ elimination in AD.

The dysfunction and death of hepatocytes in cirrhosis may affect Aβ metabolism. Because of rare reports of an overlap between cirrhosis and AD, we sought to clarify whether the Aβ-generating function of BACE and presenilin, as well as the Aβ degrading function performed by neutral endopeptidase neprilysin (NEP) [10,11], are maintained in cirrhosis. The lack of evidence for a direct correlation between cirrhosis and AD progression suggests that there are compensatory mechanisms of Aβ elimination in liver, possibly by HSCs, whose numbers are greatly increased during cirrhosis. Based on functional and phenotypic similarities between A-HSC and astrocytes, we hypothesized that A-HSC may contribute to the clearance of Aβ, similar to the activities of astrocytes in healthy brain [5].

We further hypothesized that in healthy liver, which is characterized by facilitated blood-tissue interactions, Aβ contributes to the maintenance of normal integrity and porosity of liver sinusoids through the production of nitric oxide (NO) and vascular endothelial growth factor (VEGF), which also maintains transendothelial permeability in liver and brain [12,13,14]. This hypothesis is based upon: (1) the capacity of Aβ and NO [15,16,17] to disrupt the blood-brain-barrier (BBB); (2) the functional similarity between Aβ and NO in neural cells in vivo and in vitro [15,16,18]; and, (3) the Aβ-dependent production of NO in neural tissue during AD [17].

Herein we have investigated the influence of Aβ on sinusoidal permeability and fibrotic markers in cirrhotic livers from different species, as well as in livers of the transgenic AD mice (TgAD). The latter animals are characterized by accelerated generation of Aβ and plaque burden in the brain [19,20]. This study uncovers a previously unknown activity of Aβ in regulating hepatic sinusoidal permeability and quiescence of HSC.

## 2. Materials and Methods

### 2.1. Human Liver Tissue Samples

Liver tissues for immunoblotting, PCR, and Aβ quantification by V-Plex^®^ analysis were obtained from 44 patients (21 males and 23 females) with normal liver (15 patients), with fibrosis (15 patients), and with cirrhosis (14 patients). Surgery was done because of hepatic metastases of extrahepatic tumors (19 patients), cholangiocarcinoma (4 patients), hepatocellular carcinoma (5 patients), and other diseases (4 patients), and only nonaffected or nontumorous tissue was used. Further cirrhotic liver tissue (12 patients) was obtained from explanted liver organs. Liver samples were histologically examined by a pathologist and the severity of fibrosis/cirrhosis was judged by the MELD score [21]: fibrosis 8.78 ± 3.33 and cirrhosis 15.63 ± 7.87 (see Table 1). The histological scores of tissue samples, patient’s age, gender, and assessed levels of aspartate (AST) and alanine (ALT) aminotransferase and alkaline phosphatase (AP) are listed in Table 1.

### 2.2. Animal Models

For bile duct ligation (BDL), eight-week-old male Sprague Dawley rats and C57BL/6J mice (Charles River, Sulzfeld, Germany) were separated into BDL and sham operated (SO) groups (*n* = 6 each). Ligation of the common bile duct was performed as described by Arias et al. [22]. The surgical intervention took place under halothane anesthesia. After 14 days, the rats were sacrificed and the livers were snap frozen in liquid nitrogen and stored at −80 °C. BDL in mice was done for 21 days and performed following standardized protocols published elsewhere [23,24].

Two transgenic mouse models of AD purchased from Jackson Laboratories (Bar Harbor, ME, USA) were used: (i) 6-month-old female 3×Tg-AD harboring PS1M146V, APPSwe, and tauP301L transgenes [19], and (ii) 3–4-month-old 5XFAD harboring five Familial Alzheimer’s Disease (FAD) mutations [APP K670N/M671L (Swedish) + I716V (Florida) + V717I (London) and PS1 M146L+ L286V] [20]. As control animals, C57BL/6J mice (Charles River) for 3×Tg-AD mice and WT littermates of 5XFAD mice were utilized. Mice were euthanized by CO_2_ exposure. Brains and livers were isolated, snap frozen, and stored at −80 °C.

### 2.3. Cell Culture

Mouse M1-4HSC, rat HSC-T6, and human LX-2 HSC cell lines have been described previously [25,26,27]. Human SV40-immortalized hepatic sinusoidal endothelial cells (hLSEC) were obtained from Applied Biological Materials (Richmond, BC, Canada). STR analyses of human cell lines and Mycoplasma testing of all cell lines were performed.

Astroglia-rich primary cultures (APC) were prepared from newborn C57/BL6 (Charles River) mouse brains as described elsewhere [3,4,28]. Briefly, the cells obtained from 5–7 brains of newborn littermates were mechanically dissociated, centrifuged, and plated onto cell culture flasks (1 × 10^6^ cells/75 cm^2^) in DMEM with 4.5 g/L glucose supplemented with 10% fetal calf serum, 100 µg/mL streptomycin sulphate, 100 units/mL penicillin G, and 1 µM pyruvate (Biochrom AG, Berlin, Germany) in a humidified 10% CO_2_ atmosphere at 37 °C.

M1-4HSC, HSC-T6, hLSEC, and LX-2 cells were grown in DMEM with high (4.5 g/L) glucose containing either 2% (for LX-2), 5% (for hLSEC), or 10% fetal calf serum (for M1-4HSC and HSC-T6), 1% nonessential amino acids (only for M1-HSC), 1 µM pyruvate (only for HSC-T6), 100 U/mL penicillin, and 100 µg/mL streptomycin (for hLSEC and HSC-T6, Gibco, Thermo Fisher Scientific, Darmstadt, Germany). Cells were kept at 37 °C in an atmosphere containing either 5% (for M1-4HSC and LSEC) or 10% CO_2_ (for LX-2 and HSC-T6).

### 2.4. Western Blot Analyses

Liver tissue was homogenized in ice cold lysis buffer (300 mM NaCl, 50 mM Tris, 2 mM MgCl_2_, 0.5% NP40) containing ‘Complete protease inhibitor’ (Roche, Mannheim, Germany). The total protein was determined by DC Protein assay (Bio-Rad). Proteins were fractionated by SDS/PAGE (12% acrylamide) and transferred onto PVDF membranes (EMD Millipore, Billerica, CA, USA). Membranes were blocked in 5% BSA (Albumin Fraction V, protease-free, Roth, Karlsruhe, Germany) in TBST for 1.5 h and were incubated at 4 °C overnight with respective primary antibodies (Table 2) diluted in PBS. For visualization of antibody binding, membranes were incubated with alkaline phosphatase- or Cy2/Cy5-conjugated antibodies for 3 h at room temperature. Protein bands were visualized using chemiluminescence or fluorescence detection systems (Bio-Rad, Hercules, CA, USA). For imaging and densitometric analyses, a VersaDocTM 4000 MP imaging system (Bio-Rad, Hercules, CA, USA) was used. Data were normalized to the respective densitometric values of GAPDH as loading control.

For Western blot analyses of NEP, M1-4HSC, T6-HSC cells and astrocytes were grown in 75 cm^2^ culture flasks at a density 1 × 10^6^/75 cm^2^. For Western blot analyses of α-SMA, M1-4HSC were seeded onto culture flasks at a density 1 × 10^6^ cells/75 cm^2^ and incubated for 48 h with or without 1000 pg/mL synthetic Aβ42 (Merck Millipore, Darmstadt, Germany) dissolved in standard medium and harvested for Western blot analysis. HLSEC were seeded onto culture flasks precoated with collagen (Corning, Kennebunk, ME, USA) at a density 5 × 10^5^ cells/75 cm^2^. After 12 h, adherent cells were incubated for an additional 48 h period with 1000 pg/mL synthetic Aβ42 dissolved in standard medium and harvested thereafter for Western blot analysis.

### 2.5. Real-Time qPCR of Rat Liver Tissue and Cell Cultures

RNA was extracted from rat liver tissue or mouse M1-4HSC using the RNeasy Mini Kit (Qiagen, Hilden, Germany) or mirVana miRNA Isolation Kit (Thermo Fisher Scientific), respectively. Per sample, 1 µg of RNA was transcribed to cDNA using the TaqMan Reverse Transcription Kit (Applied Biosystems, Life Technologies, Carlsbad, CA, USA); transcription was performed according to the manufacturer’s instructions. Predesigned TaqMan gene expression assays were purchased from Thermo Fisher Scientific (Table 3). RNA analyses of *α-SMA* and glial fibrillary acidic protein (*GFAP*), as well as of *GAPDH* as a housekeeping gene, were performed using the 7900 Real-Time PCR System (Applied Biosystems). The relative quantity (RQ) of target gene RNA normalized to GAPDH was calculated by the 2^−ΔΔCT^ method [29].

### 2.6. Real-Time qPCR of Human Liver Tissue

The mRNA expression was investigated by real-time qPCR using SYBR Green. Total cellular RNA was isolated with TRIzol reagent from Life Technologies (Darmstadt, Germany). Primers for human NEP/*MME* and APP, as well as BACE1, were described elsewhere [30,31]. Human PS1/*PSN1* was amplified with the primers 5′-CCT CAA CAA TGG TGT GGT TG-3′ and 5’-TTG TGA CTC CCT TTC TGT GCT-3´ and tyrosine 3-monooxygenase/tryptophan 5-monooxygenase activation protein, zeta polypeptide (*YWHAZ*) mRNA with 5′-GCA ATT ACT GAG AGA CAA CTT GAC A-3′ and 5′-TGG AAG GCC GGT TAA TTT T-3′. For quantification of the results, RNA of respective liver samples was reverse transcribed, and cDNA was serially diluted and used to create a standard curve of at least six different dilutions for each of the genes analyzed. The second derivative maximum method was used for quantification with the LightCycler software. The PCR reaction was evaluated by dissociation curve analysis, and expression values were normalized to the expression values of the housekeeping gene *YWAHZ*, encoding the tyrosine 3-monooxygenase/tryptophan 5-193 monooxygenase activation protein, zeta polypeptide.

### 2.7. Additional Information to qRT-PCR Analysis and Use of Housekeeping Gene YWAHZ

Isolated RNA were quantified at 260/280 nm with Thermo Fisher Scientific Nanodrop 2000 spectrophotometer. The absorption ratio A260 nm/A280 nm between 1.90 and 2 was taken into consideration for cDNA preparation. First strand cDNA was synthesized from 1 μg of total RNA with reverse transcriptase using the QuantiTect Reverse Transcription Kit (Qiagen) in 20 µL; 0.5 μL of the cDNA was used for amplification in a LightCycler 480 System (Roche) applying 0.5 µM of each primer for human NEP/*MME*, *APP, BACE1*, as well as PS1/*PSN1*. Real-time RT-PCR was performed in triplicates using the LightCycler ^®^ 480 SYBR Green I Master (Roche) and the specificity of the PCR reactions was confirmed by sequencing of the amplified DNA fragments (Geneart, Regensburg, Germany) and the efficiency of each PCR reaction was calculated using the serially diluted standard curve: NEP/*MME* (2.00), APP (1.97), BACE1 (2.00), PS1/PSN1 (1.99), *YWHAZ* (1.98). The housekeeping gene *YWHAZ* was chosen because of similar cp values as the genes of interest and stable expression (no statistically proven different expression) between the analyzed groups of samples [32].

### 2.8. Aβ Quantification in Cell Cultures

For analyses of Aβ effects on synthesis of α-SMA, M1-4HSC were seeded onto culture flasks at a density 1 × 10^6^ cells/75cm^2^ and incubated for 48 h with or without 1000 pg/mL synthetic Aβ42 and with or without 1 µM LBQ657 (Sigma-Aldrich, Taufkirchen, Germany) dissolved in standard medium and harvested for Western blot analysis.

For the comparison of different cell types regarding their ability to utilize Aβ42, M1-4HSC, HSC-T6, LX-2, and astroglial primary cells (APC), as well as cell lysates from M1-4HSC, were used. All cell types were seeded onto 24-well plates at a density of 100,000 cells/well. M1-4HSC cell lysates were incubated with DMEM containing 1000 pg/mL of synthetic Aβ42 and Aβ40 in the presence or absence of 5 mM EGTA for 30 or 60 min or 1 µM LBQ657 for 48 h. Aβ42 and Aβ40 were measured with the human Aβ ELISA kit (Merck Millipore, Darmstadt, Germany) according to the manufacturer’s protocol.

### 2.9. Quantification of TGF-β1 in Cell Culture Supernatants

Primary mouse HSC were isolated from C57BL/6 mice as described elsewhere [33]. At day 9, the cells were seeded onto 24-well plates at a density of 100,000 cells/well and cultured in DMEM for 24 h. The medium was then replaced with medium containing Aβ42 (1000 pg/mL) and/or LBQ657 (1 µm). TGF-β1 was measured in cell culture supernatant by ELISA using the Mouse TGF-β1 ELISA kit (LSBio, LifeSpan BioSciences, Inc., Seattle, WA, USA) according to the manufacturer’s protocol.

### 2.10. Quantification of Aβ Peptides in Liver and Brain Homogenates

In human rat and mouse livers Aβ peptides were detected by V-Plex^®^ Kit (Mesoscale, Rockville, MD) using the Aβ antibody (4G8) recognizing human and rodent Aβ40, Aβ42, and Aβ38. Mouse livers from transgenic AD mice with their respective WT controls were analyzed by Luminex assay using MILLIPLEX MAP Mouse Amyloid Beta Magnetic Bead Kit (MABMAG-83K, Merck).

### 2.11. Immunofluorescence Staining

For immunofluorescence analyses M1-4HSC, mouse primary HSC and the hLSEC line were plated in 6 cm Petri dishes (2.5 × 10^5^ cells/dish) containing coverslips. Cells were incubated with or without Aβ42 (1000 pg/mL) and/or LBQ657 (1 µM) dissolved in the respective culture medium for 48 h.

Liver and brain tissue sections (10 µm thick) and cells grown on glass cover slips were fixed in –20 °C cold methanol, washed twice in PBS, and incubated for 1 h at RT or overnight at 4 °C with primary antibodies (Table 4) diluted in PBS. After washing twice in PBS, samples were incubated with a corresponding fluorochrome-linked secondary antibody in the dark for 1 h at RT, and were washed two times for 10 min with PBS containing 0.1% Triton^®^ X-100 (Sigma). The slices and cells were then covered with Vectashield mounting medium (Vector Laboratories Burlingame, Burlingame, CA, USA) containing 4′,6 diamidino-2-phenylindole (DAPI), dried, and stored at −20 °C. As for negative controls, samples were treated with secondary antibodies only.

All immunofluorescence stainings were evaluated by fluorescence microscopy, using an Olympus BX51 Microscope (Olympus Optical Co. Europe, Hamburg, Germany). Images were acquired by the digital camera F-View II and processed by the software Analysis DOKU^®^ (Soft Imaging System GmbH, Leinfelden-Echterdingen, Germany). Primary and secondary antibodies were diluted as indicated in Table 4.

### 2.12. Collagen 1 ELISA of Cell Culture Supernatants

M1-4HSC were transferred into 24-well plates (50,000/well) and after adherence incubated with medium containing 1000 pg/mL of synthetic Aβ42 and Aβ40 for 48 h. Collagen 1 (Col-1) was analyzed in the cell culture supernatant by the mouse collagen type 1 ELISA Kit (MyBioSource, San Diego, USA).

### 2.13. Statistical Analyses

Statistical analyses were performed using GraphPad Prism Software (GraphPad Software Inc, La Jolla, CA). All data are presented as means ± SEM. (standard error of the mean). The normality was first determined by Shapiro-Wilk or d’Agostino Pearson tests. One-way ANOVA analysis with post hoc Bonferroni’s multiple comparison test vs. two-tailed Student’s *t*-tests were applied for normally distributed data sets. Nonnormally distributed data were analyzed by Kruskal-Wallis vs. Mann-Whitney test. *p* < 0.05 was considered significant.

### 2.14. Ethics Approval Statements

Experimental procedures on the use of patients’ fibrotic and normal liver tissue were performed according to the guidelines of the charitable state controlled foundation Human Tissue and Cell Research (HTCR), with the written informed patient’s consent approved by the local ethical committee of the University of Regensburg.

All animal experiments were approved by the local institutional committee of Animal Welfare in Tübingen (Regierungspräsidium Tübingen, approval number §4-14.05.2018, PH6/14), Aachen (LANUV, Recklinghausen, Germany, approval number 84-02.04.2012.A092) and Halle (Landesverwaltungsamt Halle, approval number 42502-2-1369) conducted in accordance with the German federal law regarding the protection of animals and ‘Guide for the Care and Use of Laboratory Animals’ (National Institutes of Health publication 8th Edition, 2011).

## 3. Results

**Figure 1:**
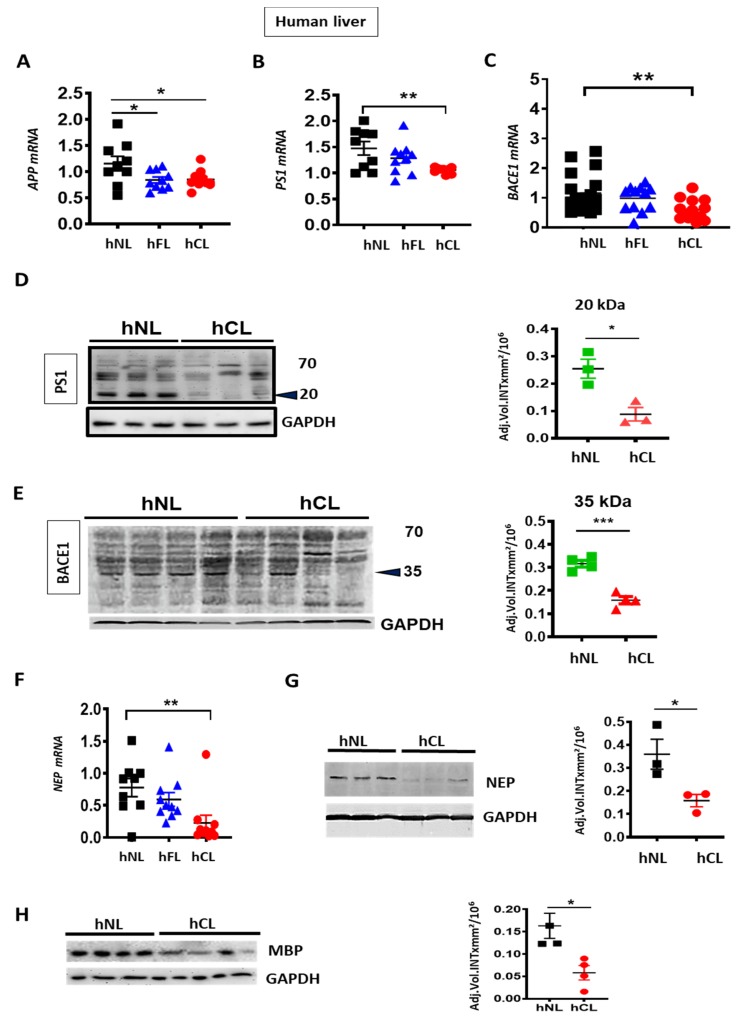
Decreased expression of amyloid precursor protein (APP), APP processing- and Aβ metabolizing enzymes in human fibrotic and cirrhotic liver. (**A**–**C**) qPCR analyses of *APP*, presenlin 1(PS1) and β-secreatase 1 (BACE1) in fibrotic (hFL, *n* = 10–12) and cirrhotic (hCL, *n* = 10–14) vs. normal human livers (hNL, *n* = 9–20). (**D**–**E**) Western blot and densitometry of PS1 and BACE1 in hNL vs. hCL (*n* = 3–4) (**F**–**G**) qPCR (*n* = 9) and Western blot (*n* = 3–4) with densitometric analysis of NEP protein and NEP mRNAin hCL vs. hNL (**H**) Western blot and densitometry of myelin basic protein (MBP) in hCL vs. hNL (*n* = 3–4). One-way ANOVA with post hoc Bonferroni’s multiple comparison in A–C, Kruskal-Wallis in 1F and Student’s *t*-tests in D, E, G and H. Means ± SEM, * *p* < 0.05, ** *p* < 0.01, *** *p* < 0.001.

We first examined the levels of APP and the enzymes involved in Aβ generation (BACE1 and PS1) and degradation (NEP) in human fibrotic (hFL) and cirrhotic liver (hCL). *APP* mRNA was down-regulated (Figure 1A) in both hFL and hCL compared to normal liver (hNL), concomitant with a decrease of PS1/*PSN1* and *BACE1* mRNA (Figure 1B–C), reflecting an overall decrease in the key drivers of Aβ generation. Furthermore, decreased BACE1 and PS1 was confirmed at the protein level by reduced expression of the enzymatically active ~20 kDa C-terminal fragment of PS1 and of the ~35 kDa BACE1 fragment in human cirrhotic liver (Figure 1D,E). On the other hand, a significant reduction of NEP/*MME* mRNA and protein (Figure 1F,G) was observed in hCL vs. hNL, which indicates a minor contribution of NEP to the Aβ decrease in human cirrhosis.

In addition, myelin basic protein (MBP), another protein known to degrade Aβ [34], was reduced in hCL (Figure 1H). This may contribute to the appearance of unmyelinated nerve fiber bundles and reduced hepatic innervation in cirrhosis [35].

**Figure 2:**
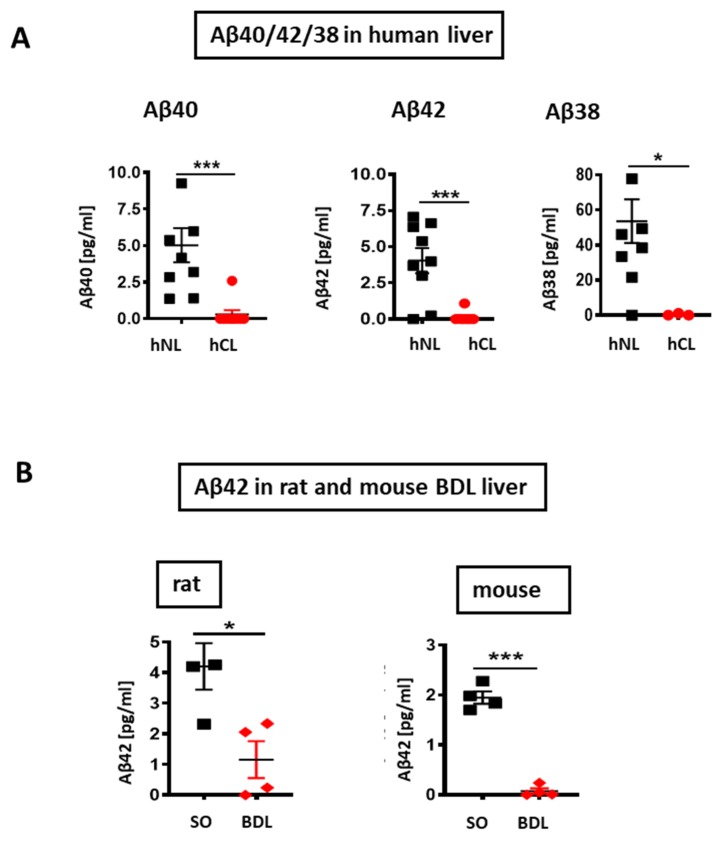
Aβ levels in cirrhotic/fibrotic and normal livers (**A**) V-PLEX analysis showing down-regulation of Aβ40, Aβ42, and Aβ38 in hCL vs. hNL (*n* = 9); (**B**) down-regulation of Aβ42 in rat BDL (*n* = 4) and in mouse BDL (*n* = 4) vs. respective SO livers. Mann-Whitney test in A and Student’s *t*-tests in B were used. Means ± SEM, * *p* < 0.05, *** *p* < 0.001.

**Figure 3:**
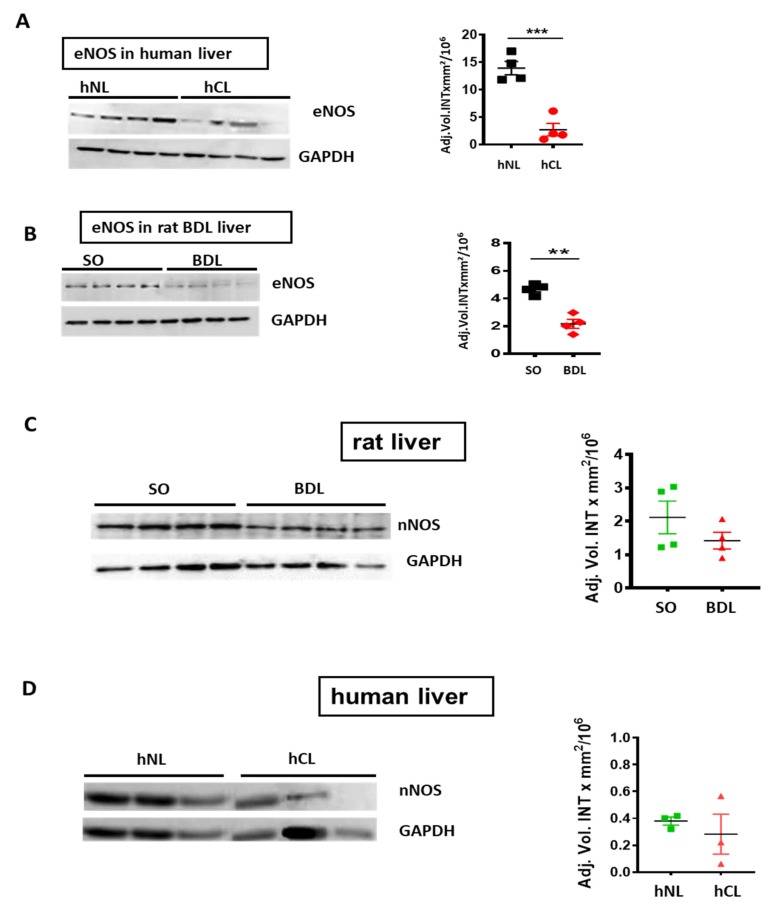
Endothelial (eNOS) and neuronal NO synthase (nNOS) in cirrhotic/fibrotic and normal livers. Western blot and densitometry of (**A**) eNOS in hCL vs. hNL(*n* = 4); (**B**) eNOS in rat BDL vs. sham operated (SO) controls (*n* = 4). (**C**) nNOS in rat BDL (*n* = 4) vs. SO (**D**) nNOS in hCL vs. hNL (*n* = 3); Student’s *t*-tests. Means ± SEM, ** *p* < 0.01, *** *p* < 0.001.

V-PLEX^®^ analysis revealed a roughly 5-, 10-, and 160-fold decrease of Aβ40/42/38 peptides respectively in hCL (Figure 2A), and a ~6-fold reduction of Aβ42 in rodent liver after BDL-induced fibrosis (Figure 2B). In addition, eNOS was down-regulated in human cirrhotic liver and in a rat bile duct ligation (BDL) model (Figure 3A,B) compared to respective controls. In both, rat BDL and hCL elicited no significant changes in the level of the neuron-specific NOS (nNOS) (Figure 3C,D).

**Figure 4:**
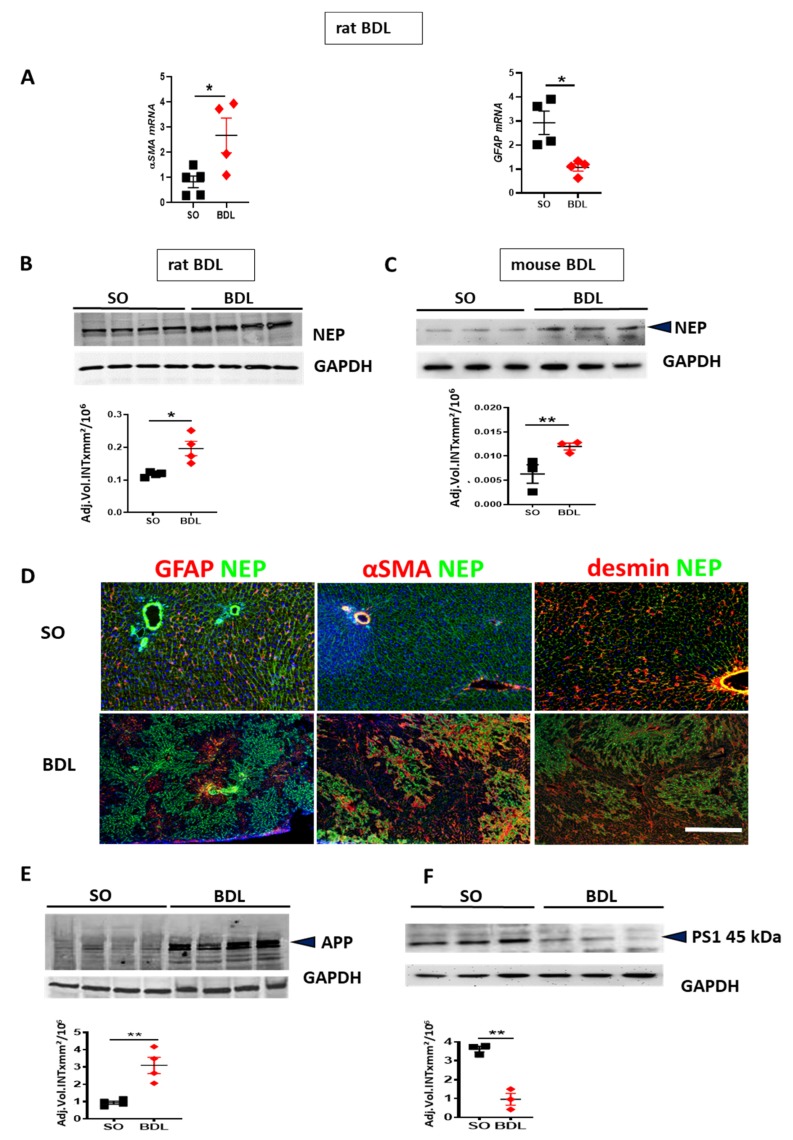
Bile duct ligation (BDL)-induced changes in proteins involved in generation and degradation of Aβ. (**A**) RT-qPCR of α-smooth muscle actin (α-SMA) and glial fibrillary acidic protein (GFAP) mRNA in the livers of BDL rats vs. sham operated (SO) controls. (**B**,**C**) Western blot and densitometry of neprilysin (NEP) in rat and mouse BDL livers vs. SO controls. (**D**) Costaining for NEP (green) and GFAP (red) (left panel), for NEP and α-SMA (red) (middle panel), for NEP and desmin (red) in (right panel) in SO (upper row) and BDL (lower row) rat liver. Cell nuclei are stained with 4′,6-Diamidin-2-phenylindol (DAPI, blue). The images are representative out of 4 animals (10 sections per animal) analyzed per group. Scale bar representative for all images in D: 200 µm. (**E**,**F**) WBs with densitometry of APP and PS1 in BDL and SO rats. Two-tailed Student’s *t*-tests. Means ± SEM (*n* = 3–6), * *p* < 0.05, ** *p* < 0.01.

In BDL rats, the development of fibrosis was confirmed by RT-qPCR analyses showing upregulation of α-SMA mRNA associated with the reduction of GFAP mRNA in BDL vs. sham operated (SO) rat livers (Figure 4A). BDL-induced fibrosis increased expression of NEP in rat (Figure 4B) and mouse livers (Figure 4C). In normal liver (upper row of Figure 4D) stained for NEP and GFAP, or α-SMA, or desmin (red fluorescence), a further marker of HSC [36], NEP was moderately expressed throughout the liver tissue. Desmin was used as a marker detectable in both activated and quiescent HSC [37]. NEP was absent from GFAP-positive HSC, but was strongly expressed in the vessels. In BDL rat liver, NEP was strongly expressed in GFAP-negative areas (lower left micrograph of Figure 4D) and in the fibrotic areas containing large population of activated α-SMA positive HSC (middle panel in Figure 4D). Most cells residing in fibrosis-unaffected areas and a large population of α-SMA positive HSC in fibrotic septae were NEP-negative (middle panel in Figure 4D). NEP was colocalized with α-SMA and desmin in fibrotic nodules (middle and right panel of Figure 4D).

**Figure 5:**
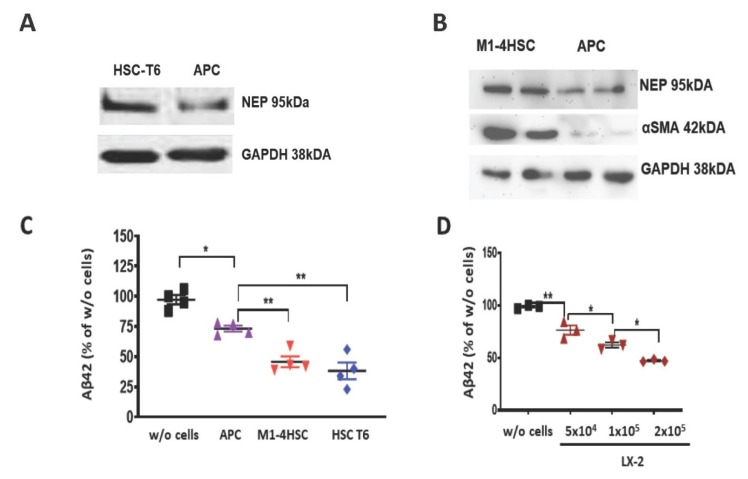
Comparison of Aβ degrading potency of rat and mouse α-SMA-positive HSC cell lines and astrocytes. (**A**,**B**) Western blots of NEP and α-SMA in lysates of HSC-T6, M1-4HSC and astroglial primary culture (APC). (**C**) Aβ42 ELISA of cell culture supernatants from APC, M1-4HSC and HSC-T6 (*n* = 4 in each group) vs. control samples without cells (w/o cells) after administration of 1000 pg/mL Aβ42. (**D**) Aβ42 ELISA of cell culture supernatants from LX-2 with increasing number of cells after administration of 1000 pg/mL Aβ42. Statistics were generated using a one way ANOVA with post hoc Bonferroni’s multiple comparison test. The data are shown as means ± SEM, * *p* < 0.05, ** *p* < 0.01, respectively.

In rat BDL livers, the amount of APP was higher than in the livers of SO animals (Figure 4E). Accumulation of APP indicates its decreased cleavage by Aβ-generating enzymes. Also, downregulation of PS1 protein (Figure 4F) reflects the failure in APP processing for efficient Aβ generation. To explore the parallels in Aβ-degrading function between HSCs and astrocytes, we first assessed the expression of the Aβ-degrading enzyme NEP by astrocytes in astroglial primary culture (APC) and rodent hepatic stellate cell lines. Rat and mouse HSC cell lines (HSC-T6 and M1-4HSC) contained more NEP than APC (Figure 5A,B). A two-fold higher uptake of Aβ42 (reflected by its decrease in cell culture supernatant) by M1-4HSC and HSC-T6 vs. APC was quantified by ELISA (Figure 5C). The amount of Aβ42 internalized by human stellate cells (cell line LX-2) increased with the number of cells in culture (Figure 5D).

**Figure 6:**
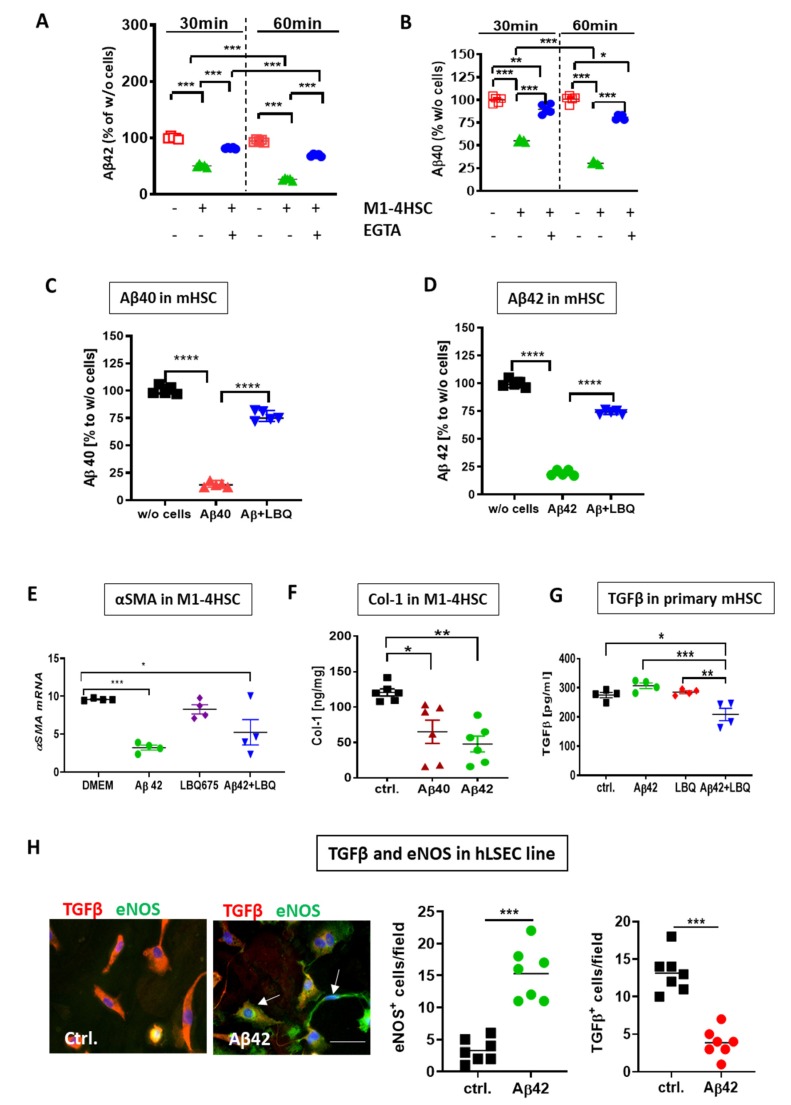
Effects of NEP inhibitors on uptake and degradation of Aβ and the expression of fibrotic markers in HSC and human liver sinusoidal endothelial cells (hLSEC). (**A**,**B**) Time-dependent degradation of Aβ42 and Aβ40 by M1-4HSC lysates (*n* = 5) assessed by ELISA 30 and 60 min after incubation with Aβ. The degradation of both Aβ fragments by M1-4HSC lysates was inhibited in the presence of EGTA (cf. M1-4HSC vs. EGTA+M1-4HSC in **A** and **B**). The concentrations of Aβ42/40 in cell-containing samples were normalized to their respective time-specific controls without cell lysates and EGTA (-/- in (**A**) and (**B**)). (**C**,**D**) Degradation of Aβ40 (**C**) and Aβ42 (**D**) exposed to the respective Aβ fragment with and without LBQ657 (1 µM) by primary mHSC (*n* = 5) assessed by ELISA. The concentrations of Aβ42/40 in cell-containing samples were normalized to their respective time-specific controls without (w/o) cells (**C**,**D**). (**E**) Expression of *α-SMA* mRNA in M1-4HSC 48h after incubation with Aβ42 and/or LBQ657. (**F**) ELISA of Col-1 in M1-4HSC incubated with Aβ40 and Aβ42; (**G**) TGF-β release by primary murine HSC in presence of Aβ and/or LBQ657 vs. control (ctrl.) assessed by ELISA; (**H**) TGF-β (red) and eNOS (green) staining in hLSEC cell line upon treatment with Aβ42. Cell nuclei are stained with DAPI; Scale bar for both images in H: 100 µm. Quantification of eNOS and TGF-β in hLSEC. Statistics were generated using a one way ANOVA with post hoc Bonferroni’s multiple comparison test in (**A**–**G**) and two-tailed Student’s *t*-test in (**H**). The data are shown as means ± SEM, * *p*<0.05, ** *p* < 0.01, *** *p* < 0.001, **** *p* < 0.0001, respectively.

To investigate whether Aβ simply accumulates or undergoes enzymatic degradation by the cells, the activities of zinc-dependent Aβ-degrading enzymes known to be present in HSC were blocked by 5 mM EGTA in M1-4HSC cell lysates. After 30 and 60 min of incubation, the level of Aβ was reduced to 50% and 25% respectively, compared to the initial level in control samples without cell lysates (Figure 6A,B). This confirmed the enzymatic degradation of Aβ by M1-4HSC rather than its nonspecific loss that can occur in cell lysates [38]. The use of the specific NEP inhibitor LBQ657 established that enzymatic Aβ metabolism in HSC can be ascribed primarily to the activity of NEP. In the presence of LBQ657, the utilization of Aβ40 and Aβ42 from the medium was inhibited by 75% compared to samples without LBQ657, as evidenced by the increased level of Aβ peptides in the supernatants (Figure 6C,D).

To test whether Aβ suppresses the activation of HSC, we examined the expression of the fibrotic hallmark proteins α-SMA, TGF-β, and collagen I (Col-1 [39]) in HSCs. Aβ markedly reduced *α-SMA* mRNA in M1-4HSC (Figure 6E). Furthermore, *α-SMA* mRNA decreased in M1-4HSC after incubation of cells with Aβ and LBQ657 (Figure 6E). Both Aβ42 and Aβ40 reduced Col-1 production (Figure 6F) in M1-4HSC. In primary mHSC, Aβ42 decreased the release of TGF-β into the culture supernatant only upon simultaneous incubation of cells with LBQ657 (Figure 6G). Aβ applied alone did not decrease TGF-β (Figure 6G, cf. ctrl. Vs. Aβ42). This can be ascribed to Aβ’s rapid degradation by primary HSC (in contrast to M1-4HSC), whereas in combination with an agent preventing its degradation, LBQ657, Aβ showed efficacious downregulation of TGF-β-release by HSC.

The increasing number of data shows that human LSEC constitutively produce high levels of TGF-β [40] that, in turn, activates HSC. The antifibrotic effects of Aβ were further confirmed by the inhibition of TGF-β production in immortalized human liver sinusoidal endothelial cell line (hLSEC), as shown by immunofluorescence analysis and quantification of TGF-β- and eNOS-positive cells (Figure 6H).

To explore the role of Aβ on the factors increasing the permeability of liver endothelial cells, its influence on the expression of eNOS, the marker of sinusoidal permeability, in immortalized hLSEC was evaluated. The decrease of TGF-β correlated inversely with eNOS in hLSEC, as demonstrated by an intense staining of eNOS in cells barely expressing TGF-β by immunofluorescence images (arrows in Figure 6H) and quantification of eNOS- vs. TGF-β-positive cells (Figure 6H).

**Figure 7:**
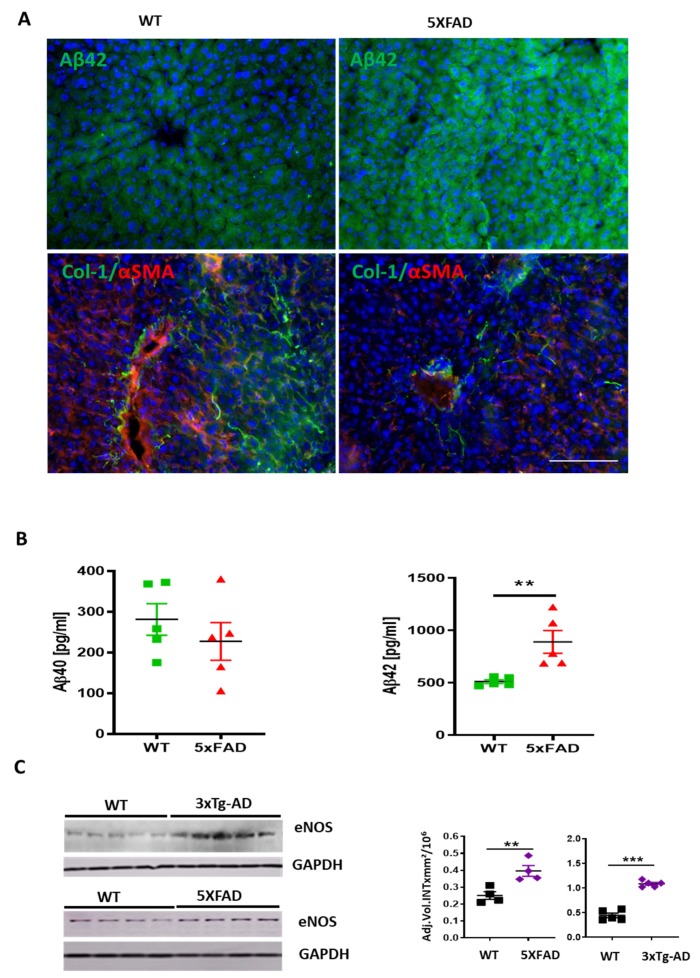
Up-regulation of Aβ and eNOS in the liver of 5xFAD and 3×Tg-AD mice. (**A**) Immune fluorescence analysis showing Aβ42 immunoreaction (green in upper row), α-SMA (red) and Col-1(green in lower row) in 5XFAD vs. WT mouse sections. Scale bar of 100 µm applies to all images in A. (**B**) Multiplex analysis of soluble murine Aβ40 and Aβ42 in liver homogenates of 5XFAD mice vs. WT controls. (**C**) Western blot analysis and densitometry of eNOS in 5xFAD and 3×Tg-AD mice vs. WT age matched controls (*n* = 4–5). Two-tailed Student’s *t*-test. The data are shown as means ± SEM, ** *p*<0.01, *** *p* < 0.001.

We assessed the expression of eNOS in the livers of 3×Tg-AD and 5XFAD transgenic mice, which have been previously well-characterized as models of AD [19,20]. In these mice, Aβ production in the brain and its systemic level were increased [41,42]. Immunofluorescence of Aβ42 (Figure 7A) and multiplex analysis of Aβ40 and Aβ42 (Figure 7B) displayed stronger immunoreaction of Aβ42 in liver sections and homogenates of 5XFAD compared to the wild type (WT) mice. In addition, a decrease in the expression of Col-1 and α-SMA was observed in 5XFAD mice (Figure 7A). As a reflection of Aβ’s influence on sinusoidal permeability, there were 1.6- and 2.2-fold increases in eNOS detected by WB in livers of 5XFAD and 3×Tg-AD mice, respectively, compared to corresponding age matched nontransgenic controls (Figure 7C).

**Figure 8:**
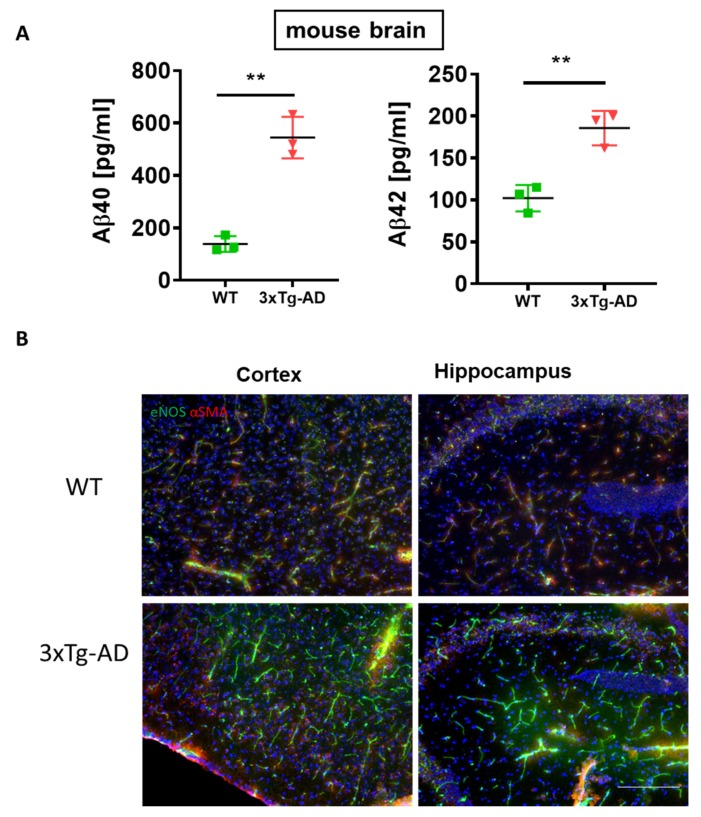
Correlation between generation of Aβ42 and eNOS in the brain of 3×Tg-AD mice. (**A**) multiplex analysis of soluble Aβ40 and Aβ42 in 3×Tg-AD mouse brain homogenates vs. WT controls. Two-tailed Student’s *t*-test. The data are shown as means ± SEM, ** *p*<0.01. (**B**) Increased expression of eNOS in the cortex and hippocampus of 3×Tg-AD mice vs. wild type (WT) controls. Brain sections taken from *n* = 3 mice per group were stained with eNOS (green) and α-SMA (red). Please note the increased expression of eNOS in the cortex and hippocampus of 3×Tg-AD mice. Scale bar of 200 µm applies to all images in B.

The brain of 3×Tg-AD mice exhibits high content of soluble Aβ40, Aβ42 concomitant with increased expression of eNOS (Figure 8A,B). High brain levels of Aβ simultaneous to upregulation of eNOS have been reported in another transgenic model of AD with impaired BBB function [43]. This, together with our results, may hint at a blood-tissue modulating function of Aβ, which, in the brain, leads to the impairment of the BBB, while in the liver, its high content assures the facilitated blood-liver exchange supporting normal liver function.

Altogether, our data strongly supports the notion that Aβ plays an important role in the liver, maintaining sinusoidal permeability while exhibiting antifibrotic activity.

## 4. Discussion

Herein we describe the down-regulation of Aβ peptides in human and rodent fibrotic and cirrhotic livers and demonstrate Aβ-dependent regulation of liver sinusoidal permeability markers using different in vitro and in vivo models.

Activation and contraction of HSC in cirrhosis leads to increased extracellular matrix protein production resulting in defenestration of liver sinusoids [44] which limit blood-liver exchange and hepatic flow. We demonstrate that Aβ promotes the maintenance of a quiescent phenotype of HSC, as evidenced by its suppressive effects on α-SMA in activated HSC. Notably, in human cerebrovascular smooth muscle cells, Aβ induces the degradation of α-SMA [45].

Liver perfusion is regulated by NO, a powerful vasodilator produced in hepatocytes and endothelial cells [46]. The Aβ-induced inactivation of HSC reflected in downregulation of α-SMA and Col-1 and the increased production of NO in LSEC are similar to the effects of NO in vivo. Furthermore, HSC-targeted nanoparticle delivery of NO blocks hepatic Col-1 and α-SMA expression in rats with fibrosis and portal hypertension [47].

Our data suggest that Aβ exerts its antifibrotic function by both autocrine and paracrine effects on HSCs and LSECs: Aβ-suppressed TGF-β release and elevated NO-production by LSEC may inhibit Col-1 and α-SMA generation in HSC. The coordinated actions of Aβ and NO in the liver can be deduced from the functional link between them in neurochemical studies [15,16,17,18], and in our in vitro and in vivo studies demonstrating increased intracellular amounts of eNOS in Aβ-treated hLSEC, from high levels of Aβ and eNOS in the liver of TgAD mice, as well as from their simultaneous reduction in human and rodent cirrhosis. These results are consistent with the previously reported influence of Aβ fibrils on the generation of stable NO metabolites in the brain [48]. The causative relationship between the loss of Aβ and NO/eNOS in human and rodent cirrhotic/fibrotic liver shown here can be concluded from the established fact that NO positively regulates the expression of key enzymes of Aβ generation, BACE1, and PS1 [49]. Accordingly, the decrease of BACE1, PS1 and Aβ in human cirrhotic and rodent fibrotic liver explains the cross talk between Aβ- and NO-producing systems in healthy and diseased liver.

Another finding of this study is that cirrhosis down-regulates MBP, the main component of myelin sheaths which are significantly decreased during cirrhosis [35]. In vitro, MBP, together with Aβ and proinflammatory cytokines, strongly stimulate the expression of functional NO synthase-2 and the production of NO via activation of inducible NOS in adult human astrocytes [18]. Thus, it is likely that endothelial cell dysfunction during cirrhosis, characterized by poor permeability of liver sinusoids, is most likely partially caused by decreased MBP-stimulated production of NO.

Low levels of NEP and MBP in human cirrhosis may also decrease the hepatic clearance of Aβ delivered by the blood, leading to its increased plasma levels in cirrhotic patients [50]. The physiological significance of the rise of NEP in BDL is poorly understood. In view of the capacity of NEP to degrade Aβ, its upregulation in the BDL model of cirrhosis may exaggerate injury already promoted by low levels of PS1 and BACE1. From a physiological point of view, in the condition of low activity of Aβ -generating enzymes, a further decrease of Aβ by NEP degradation in BDL seems meaningless. High portal pressure in the BDL model is mainly ascribed to an excess of angiotensin (Ang) II generated from Ang I and catalyzed by ACE [51]. The contribution of NEP to increased portal pressure was disproved by vasoconstrictor effects of thiorphan, the specific inhibitor of NEP [52]. The increase of NEP in BDL liver could be a counter-regulatory mechanism contributing to generation of Ang-(1–7), a vasorelaxant that is increased in BDL to counteract the vasoconstrictor effects of ACE and Ang II [51].

**Figure 9:**
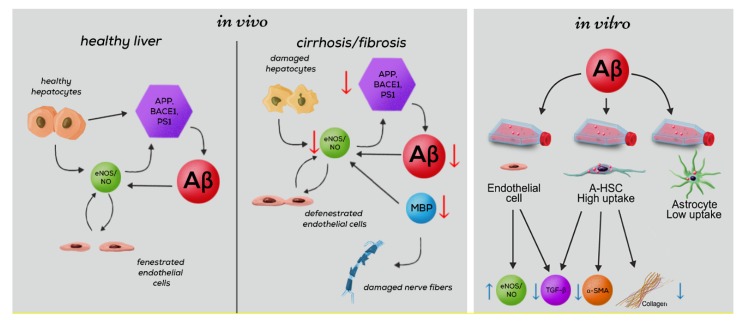
Influence of Aβ on the liver-specific functions associated with the permeability of liver sinusoids. Aβ-related activities in normal liver (left panel) and changes in cirrhosis affected liver (middle panel) based on in vivo results of this study. Summarized results showing the decrease of Aβ and proteins involved in its generation (APP, BACE-1, PS1) and degradation (NEP, MBP) during cirrhosis. Damage to nerve fibers through impaired production of MBP. Influence of impaired Aβ generation on the permeability of liver sinusoids via reduced eNOS. Summary of in-vitro results (right panel): Aβ-associated activities of cultured liver endothelial cells, activated HSC (A-HSC) and astrocytes. Schematic presentation of Aβ (pink circles) uptake by HSC and astrocytes. In our experiments, a greater capacity for internalization of Aβ by A-HSC vs. astrocytes was shown. Antifibrotic effects of Aβ on HSC and endothelial cells: decreased synthesis of TGF-β, α-SMA and collagen 1 by A-HSC; decreased production of TGF-β and increased synthesis of eNOS by liver endothelial cells.

Our findings (as outlined in Figure 9) highlight Aβ as a coordinator of multiple signals and interactions between hepatocytes, HSC, and LSEC, which are summarized in the following scenario: Healthy hepatocytes generate large amounts of Aβ via amyloidogenic proteolysis of APP by BACE and PS1 [6,53]. Aβ released into the extracellular space prevents fibrosis and increases vascular permeability. The importance of Aβ for liver-specific functions is particularly evident from its reduction in cirrhosis. During cirrhosis, the decreased expression of APP and its cleaving enzymes BACE1 and PS1, which are regulated by NO [49], results in a dramatic intrahepatic decrease of Aβ. In cirrhosis, impaired synthesis of MBP, which mimics the effects of Aβ in increasing the production of NO [15,16,17,18] may further reduce sinusoidal permeability and damage hepatic nerves. Aβ controls the phenotype of HSC and LSEC by suppressing α-SMA, TGF-β, and Col-1, and by reverting activated HSC to quiescence. The Aβ-mediated cross talk between HSC and LSEC further promotes the permeability of LSEC via upregulation of eNOS and decreased Col-1 production by HSC.

The clinical implications of our findings can be summarized as follows: Because Aβ may have opposing effects in brain and liver, efforts to lower its accumulation in the brain could have adverse effects on the liver. This would need to be considered in any clinical trials utilizing Aβ antibodies, as well as BACE and γ-secretase inhibitors, which may influence the systemic Aβ concentration [54,55]. Therefore, more tailored treatment approaches, such as vaccination against brain-specific forms of Aβ, may be required, especially in patients with concurrent liver disease. One of these strategies targeting a posttranslationally modified Aβ [56] is currently under clinical evaluation.

Furthermore, our data suggest that Aβ constructs that specifically bind to HSC, either alone or in combination with an approach targeting IFNγ [57] and/or NO [46], might be a potential therapeutic option for the treatment of advanced stages of cirrhosis. These three target molecules are known to regulate the permeability of blood-tissue interfaces [58,59,60].

## Figures and Tables

**Table 1:** Histological scoring of human liver samples.

Human Liver Tissue Sample ^a^	Histological Score (0/1/2/3/4)^a^	MELD Score ^b,c^	Steatosis (0/1/2/3) ^d^	Gender (m/f)	Age	AST [U/L]	ALT	AP [U/L]
normal (*n* = 15)	(15/0/0/0/0)	7.3 ± 1.3	(15/0/0/0/0)	(4/11)	57.1 ± 12.8	30.9 ± 13.7	27.6 ± 15.5	111.3 ± 59.2
fibrosis (*n* = 15)	(0/1/6/8/0)	8.8 ± 3.3	(10/4//1/0)	(12/3)	63.0 ± 11.9	48.5 ± 42.4	46.7 ± 26.9	200.9 ± 190.8
cirrhosis (*n* = 14)	(0/0/0/0/14)	15.6 ± 7.3	(10/1/3)	(5/9)	51.6 ± 7.9	122.0±139.8	66.7 ± 80.4	211.1 ± 117.1

Note: Tissue samples were characterized as follows: (a) Histological score was defined as no fibrosis (0), zone 3 perisinusoidal/pericellular fibrosis, focally or extensively present (1), zone 3 perisinusoidal/pericellular fibrosis with focal or extensive periportal fibrosis (2), zone 3 perisinusoidal/pericellular fibrosis and portal fibrosis with focal or extensive bridging fibrosis (3) and cirrhosis (4); (b) The MELD (Model of End Stage Liver Disease) scores were calculated using blood levels of creatinine, INR and total bilirubin [21]; (c) mean ± SD; d, Grade of steatosis was scored as <5% steatosis (0), 5 to 33% steatosis (1), >33 to 66% steatosis (2), and >66% steatosis (3). Normal ranges for selected parameters are: AST, 5–40 U/L; ALT, 5–40 U/L; AP, 35–130 U/L.

**Table 2:** Primary polyclonal (pab) and monoclonal (mab) antibodies used in Western blot analyses.

Antibody	Cat. No.	Dilution	Supplier
APP, rabbit pab	SAB3500274	1:1000	Sigma-Aldrich, Taufkirchen, Germany
α-SMA, rabbit pab	ab5694	1:1000	Abcam, Cambridge, UK
eNOS, rabbit pab	ab95254	1:500	Abcam
Neprilysin, rabbit mab, clone EPR2997	ab79423	1:1000	Abcam
MBP, mouse mab clone MBP101	ab62631	1:500	Abcam
nNOS, rabbit pab	ab5586	1:1000	Abcam
PS1, rabbit pab	PA5-20376	1:750	Thermo Fisher Scientific, MA, USA
BACE1, rabbit pab	PA1-757	1:1000	Thermo Fisher Scientific
GAPDH, mouse mab, clone 6C5	MAB374	1:500	EMD Millipore, Billerica

**Table 3:** Gene expression assays used for rat liver and mouse M1-4HSC RNA analysis.

Target Gene	Gene Symbol	RefSeq	Assay ID
α-SMA	*Acta2*	NM_031004.2	Rn01759928_g1
GFAP	*Gfap*	NM_017009.2	Rn00566603_m1
α-SMA	*Acta2*	NM_007392.3	Mm01204962_gH
GAPDH	*Gapdh*	NM_008084.3/NM_001289726.1	Mm99999915_g1

**Table 4:** Primary and secondary antibodies used in double immune labelling studies.

Antibody	Cat. No	Dilution	Supplier
Desmin mouse, mab, clone DE-U-10	D 1033	1:80	Sigma-Aldrich, Taufkirchen, Germany
GFAP mouse, mab, clone GF12.24	GF 12.24	1:10	ProGen, Heidelberg, Germany
GFAP rabbit, pab	Z 0334	1:300	Dako, Glostrup, Denmark
NEP rabbit, mab, clone EPR2997	ab 79423	1:200	Abcam, Cambridge, UK
α-SMA mouse, mab, clone ASM-1	61001	1:100	ProGen
eNOS, rabbit pab	ab 95254	1:150	Abcam
Amyloid β42, mab clone D3E10	12843	1:500	Cell Signaling, Frankfurt am Main, Germany
FITC-conjugated goat antirabbit IgG	111-095-144	1:100	Dianova, Hamburg, Germany
FITC-conjugated goat antimouse IgG	115-095-003	1:100	Dianova
Cy3-conjugated goat antimouse IgG	111-165-144	1:800	Dianova
Cy3-conjugated goat antirabbit IgG	115-165-166	1:800	Dianova
